# Time-series MODIS Image-based Retrieval and Distribution Analysis of Total Suspended Matter Concentrations in Lake Taihu (China)

**DOI:** 10.3390/ijerph7093545

**Published:** 2010-09-27

**Authors:** Yuchao Zhang, Shan Lin, Jianping Liu, Xin Qian, Yi Ge

**Affiliations:** State Key Laboratory of Pollution Control and Resource Reuse, School of the Environment, Nanjing University, Nanjing 210093, China; E-Mails: zhangych@nju.edu.cn (Y.Z.); sueann7@163.com (S.L.); vera_1203@163.com (J.L.); ge.ellie@gmail.com (Y.G.)

**Keywords:** total suspended matter, MODIS, Lake Taihu, common empirical algorithms

## Abstract

Although there has been considerable effort to use remotely sensed images to provide synoptic maps of total suspended matter (TSM), there are limited studies on universal TSM retrieval models. In this paper, we have developed a TSM retrieval model for Lake Taihu using TSM concentrations measured *in situ* and a time series of quasi-synchronous MODIS 250 m images from 2005. After simple geometric and atmospheric correction, we found a significant relationship (R = 0.8736, N = 166) between *in situ* measured TSM concentrations and MODIS band normalization difference of band 3 and band 1. From this, we retrieved TSM concentrations in eight regions of Lake Taihu in 2007 and analyzed the characteristic distribution and variation of TSM. Synoptic maps of model-estimated TSM of 2007 showed clear geographical and seasonal variations. TSM in Central Lake and Southern Lakeshore were consistently higher than in other regions, while TSM in East Taihu was generally the lowest among the regions throughout the year. Furthermore, a wide range of TSM concentrations appeared from winter to summer. TSM in winter could be several times that in summer.

## 1. Introduction

Total suspended matter (TSM) plays a key role in water quality evaluation, especially of inland waters (e.g., lakes, reservoirs). It determines the transparency of water and can also serve as a transport medium for various pollutants [1], e.g., heavy metals. The degradation of water quality due to high TSM concentrations has become a major global environmental problem. Therefore, obtaining the spatial and temporal distribution of TSM is necessary for understanding, managing and protecting aquatic ecosystems.

With the development of remote sensing technology, satellite estimates of water quality significantly complement conventional monitoring techniques and have found widespread applications. Remotely sensed monitoring of TSM started in 1974 using ERTSA data [2]. Since then, data remotely sensed from airborne and space-borne platforms have been widely used to estimate TSM in estuaries [3], bays [4], reservoirs [5], lakes [6] and coastal waters [7]. Estimating water quality from remote sensing has four main advantages: the ability to cover large areas, rapid results, low cost, and convenience for dynamic monitoring.

For water quality management of large lakes like Lake Taihu, a severely eutrophic lake, it is essential to quickly and accurately understand the dynamic variations in water quality. TSM monitoring from remote sensing in Lake Taihu has been studied for many years, and the methodologies used can be divided into three types: (1) analytical methods, also referred to as bio-optical algorithms, in which reflectance spectra are simulated by theoretical formulas based on the specific inherent optical properties of substances in the water [8,9]; (2) empirical methods, based on linear or nonlinear regression functions between remote sensing data (possibly with single band or combination of bands) and water quality parameters sampled from the water body simultaneously, or almost simultaneously, with the over-flight of sensors [10]; (3) semi-empirical or semi-analytical methods, being integrations of analytical and empirical methods [11]. The analytical method relies on many complicated and sometimes uncertain parameters, so it has not been used as widely up to now [8], and the empirical and semi-empirical methods have proven more practical. However, models developed using these two methods are generally based on a single day or different seasons [12], and are generally not suitable for other times. Although the applicability of seasonal models is better than one-day models, they still cannot provide a consistent and holistic representation of variation trends of TSM concentrations. Little information is available in the existing literature for establishing a universal model without time limitations, for which multi-temporal satellite images are necessary and helpful. Due to limitations such as temporal, spatial and spectral resolution, atmospheric correction methods, and time lag between field sampling and satellite overflight, time-series satellite image-based retrieval of TSM in heavy polluted case II waters still remains a challenge.

In this paper, Lake Taihu was selected as the study area, and a common yearly-scaled TSM retrieval model is developed. Synoptic maps of TSM in 2007 are obtained using this model, and the characteristic temporal and spatial variability of TSM is analyzed. In addition, considering that atmospheric correction is essential, especially for multi-temporal MODIS images, a simple but effective image-based relative radiometric normalization method is presented. The significance of this study is that it is the first attempt to establish a common TSM retrieval model for practical application in Lake Taihu.

## 2. Study Area

**Figure 1.**
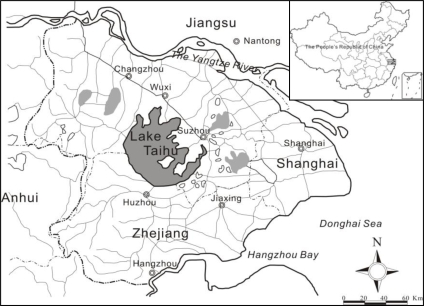
Catchment area of Taihu Basin.

Lake Taihu is located in eastern China between 30° 55′ 40″ N and 31° 32′ 58″ N, and 119° 52′ 32″ E and 120° 36′ 10″ E. It is the third largest freshwater lake in China, with a total water area of about 2,338.11 km^2^. It is a shallow lake, with mean depth 1.9 m, and maximum depth 2.6 m at a water surface elevation of 3.0 m above sea level. The Lake Taihu drainage basin (Figure 1) is the most industrialized area in China with high population density and urbanization. Lake Taihu is important for water supply, flood control, tourism and recreation, shipping and aquaculture. With the increasing pollution from both urban and rural areas surrounding the Lake Taihu Basin over the past two decades, eutrophication and algal blooms (*Microcystis* spp.) have occurred frequently, which badly affects the function of the lake as a drinking water supply. More efficient water quality monitoring of the lake is urgently needed [13].

## 3. Methods

### 3.1. Field Survey

**Figure 2.**
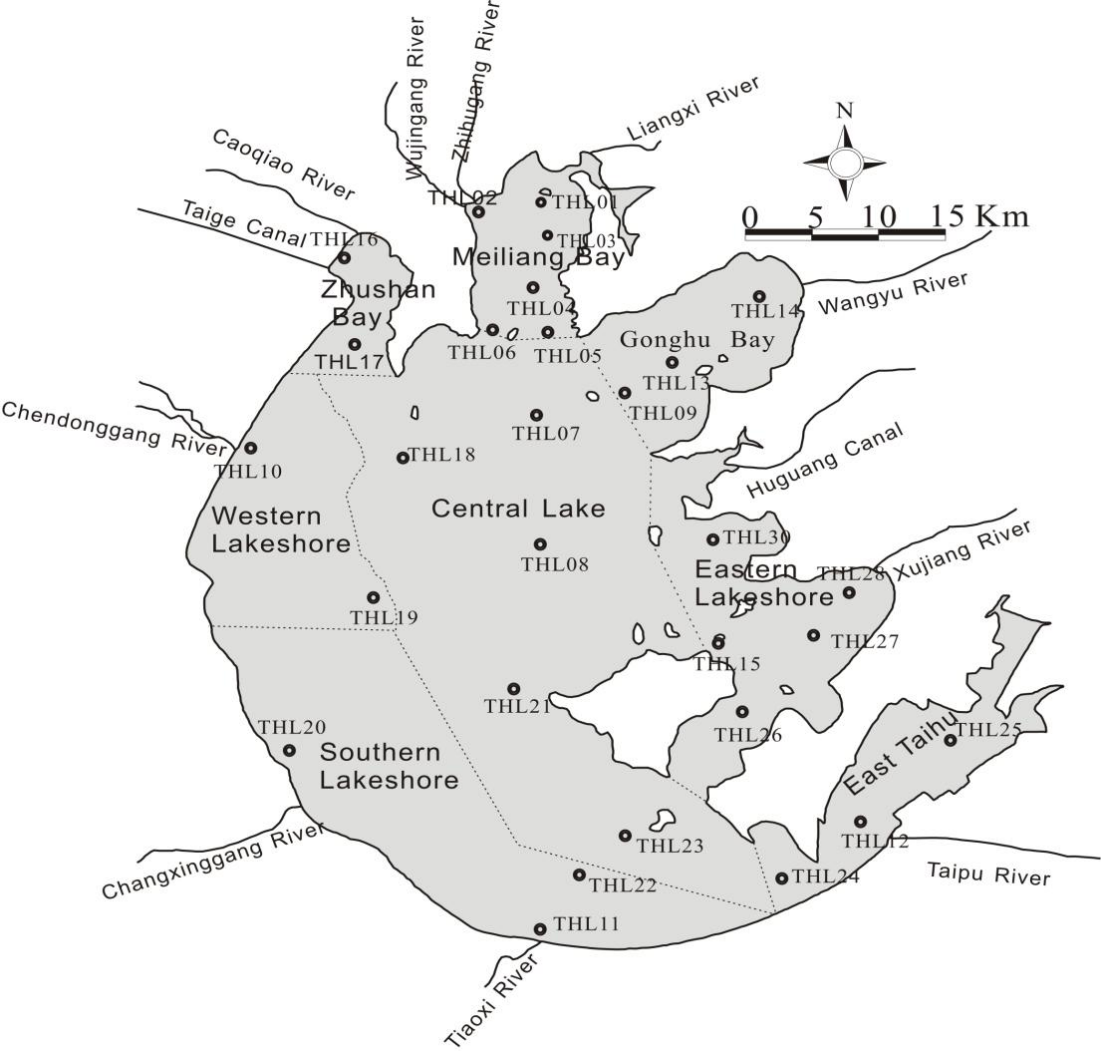
Distribution of the 28 sampling sites in Lake Taihu (THL01–28).

TSM *in situ* measurements were carried out once a month in 2005 by the Taihu Ecosystem Research and Field Observation Station, Chinese Academy of Sciences (TERFOS, CAS). Twenty-eight sampling sites were selected, which were considered representative of surface distributions of TSM encompassing the whole of Lake Taihu. February, May, August and November water samples were collected at all 28 sampling sites, while in the other eight months the field survey was only conducted at 13 sites, mainly located in the north part of Lake Taihu. The distribution of the 28 sampling stations in Lake Taihu is shown in Figure 2. Table 1 shows the detailed information of the field measurements during 2005. Furthermore, there were 16 sampling stations from 13 November 2003, located in the north of Lake Taihu, which were also monitored by TERFOS. These are used for validation.

TSM concentrations were measured by filtering the water samples (taken from 0.5 m below the water surface) on Whatman Grade GF/F filters, which were dried and weighed on an electronic balance.

### 3.2. MODIS Imagery

MODIS images provide high radiometric sensitivity (12 bit) in 36 spectral bands from 0.4 μm to 14.4 μm in wavelength. The spatial resolution is 250 m for bands 1–2, 500 m for bands 3–7, and 1,000 m for bands 8–36. As mentioned above, numerous studies have demonstrated that MODIS has the potential for use in water quality monitoring. In particular, the MODIS 250 m and 500 m bands, centered at 469 nm (b3), 555 nm (b4), 645 nm (b1) and 858 nm (b2), can provide sufficient sensitivity for water applications [14]. In this study these four bands were utilized for TSM retrieval.

MODIS-Terra level 1B calibrated radiance products, with spatial resolutions of 250 m and 500 m, covering the region of interest in the middle November of 2003 and the whole of 2005 and 2007 were downloaded via the web interface of the Level 1 and Atmosphere Archive and Distribution System (LAADS) of the National Aeronautics and Space Administration (NASA). MODIS images from 2005 were obtained for days corresponding to field measurements of TSM, and the time difference between the field measurements and MODIS images was no more than three days. Because of cloud coverage over the target no appropriate images were available for March and June of 2005. Two cloud-free MODIS images from every month (except June) in 2007, were downloaded to analyze the distribution variation of TSM in Lake Taihu.

(1) Geometric correction

Each image was geo-referenced based on the WGS 84 UTM zone 51 North coordinate system. The 500 m resolution images were resized to 250 m using the nearest neighbor resampling method during geo-referencing.

(2) Atmospheric correction

Besides the changes in target reflectance, changes in satellite sensor calibration, differences in illumination and observation angles, and variations in atmospheric effects can induce variation in a sensor’s response to a given target over time. Therefore, to detect the actual changes in ground target from the total signal measured at the spacecraft sensor, atmospheric correction is necessary. Two types of atmospheric corrections, absolute correction and relative correction, are commonly employed to normalize remotely sensed images for time-series inter-comparison [15–17]. Absolute correction is aimed towards extracting the absolute reflectance of scene targets at the surface of the earth, requiring the input of simultaneous atmospheric properties and sensor calibration. These are difficult to acquire in many cases, especially in historic data [18]. Relative correction is aimed towards reducing atmospheric and other unexpected variations among multiple images by adjusting the radiometric properties of target images to match a base image [19] and setting the multi-temporal images into a common scale without extra parameters from other measurements. Generally, relative correction methods are simpler than absolute correction methods [20], and existing studies have shown that relative normalization is the most critical step in obtaining temporal stability in a series of images [21,22].

A variety of relative radiometric correction techniques have been developed [16], most of which assume that radiometric relationships between the target image and the base image are linear [16,20,23]. One relative correction method, the Pseudo-Invariant Features method (PIFs), is most commonly used. Schott *et al*. [15] developed this method using spectrally pseudo-invariant features, such as impervious roads, roof tops and parking lots. Given that the pseudo-invariant features were selected by subjective visual inspection, Du [23] developed an improved method for PIF selection, in which PIFs were selected via principal component analysis (PCA) using the scatter plots of two images, and quality control of the normalized image was conducted to ensure the accuracy of the radiometric correction. In addition, Chen [24] proposed another simple relative radiometric correction method, the Temporally Invariant Cluster (TIC) method. In this method, rigorous selection of invariant surface features is not necessary, and the line intersecting all TIC centers is the normalization regression line. Integrating the above two methods we employed two zones as the pseudo-invariant features according to the procedure of Du [23], and the normalization regression line was the line intersecting the strictly selected the centers of these two zones, based on the method of Chen [24]. Assumptions of these two methods are that there are at least two PIFs with spectral characteristics that can be significantly distinguished from each other and other variant pixels, and that variations in PIFs involved in the correction procedure are linear and spatially homogeneous during the period [23,24]. Therefore, one set of the PIFs is defined by:

$$\text{PIF}\;\;\;\text{set}=\left\{1\leq \frac{R_{\text{band}\;2}}{R_{\text{band}\;1}}\leq 1.5\;\;\;\text{and}\;\;\;R_{\text{band}\;2}\geq 30\right\}$$

where *R_band1_* and *R_band2_* are the radiance of band 1 and band 2 respectively. These PIFs are mainly situated in the new industrial zone around Taihu Lake, such as Changzhou National Hi-tech District, Wuxi Economic District and Suzhou National Hi-tech District. The other set of PIFs is located in clean sea, where water quality is good and little influenced by TSM. Following the normalization procedure of Du, these two screened PIFs sets were achieved. After identifying the PIFs centers in the density map, the normalization regression line is obtained as that intersecting these two PIFs centers. The last step is to recalculate the target image pixel values based on the normalization regression function. Now, the target image has been normalized to the scale of the base image [24]. According to the method mentioned above, the image of January 2005 was randomly selected as the base image representing the common scale. Then the image from November 2003, 10 images from 2005 and 23 images from 2007 were all normalized to this common scale.

**Figure 3.**
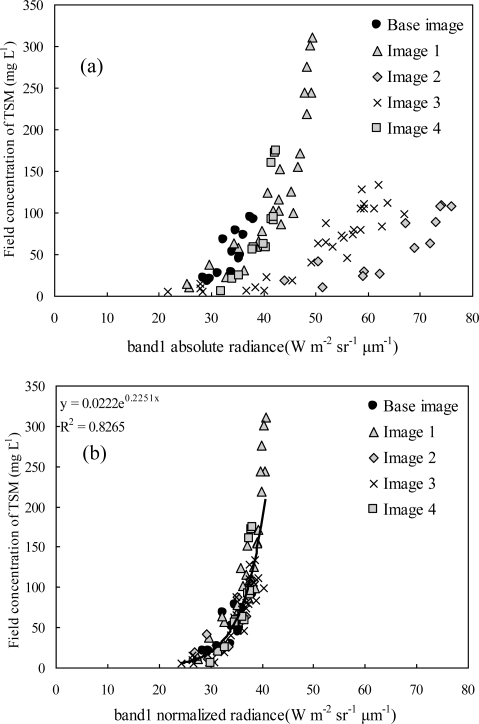
(**a**) Relationship between TSM concentrations and band1 radiance before the atmospheric correction; (**b**) Relationship between TSM concentrations and band1 radiance after the atmospheric correction (base: January 2005, Taihu Lake area. Images 1–4: February, May, September and December, 2005, respectively).

Figure 3 shows the relationship between TSM concentrations and band 1 radiance before and after the atmospheric correction, from which we can see that after the atmospheric correction the other images (images 1–4) corrected to the scale of the base image (January 2005).

### 3.3. Retrieval of Total Suspended Matter Concentrations from MODIS Data

In total, there were 166 pairs of radiometric normalized MODIS radiance data and *in situ* measured TSM concentration data in 2005. All 166 data pairs were used for the development of the TSM retrieval model, and another 16 data pairs from 2003 were used for model validation.

Instead of using a sophisticated optical model, simple linear and non-linear models (e.g., Logarithmic, Inverse, Quadratic, Cubic, Power, Compound, S-curve, Growth and Exponential functions) were used to correlate TSM concentration (mg L^−1^) to atmospherically corrected MODIS band data. This included single band (band 1–4) and band combinations (band difference, band ratio and band normalized difference). The best fitting model was selected to map the temporal and spatial variability of TSM in 2007.

## 4. Results

**Figure 4.**
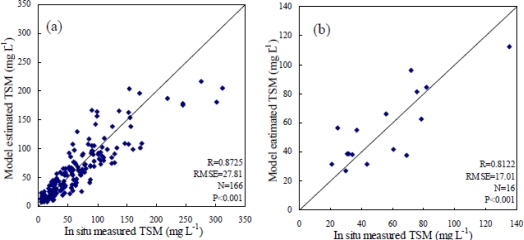
Comparison of *in situ* measured TSM and estimated TSM from the band ratio Quadratic model listed in Table 2 using (**a**) 166 data pairs of 2005 for model development; (**b**) 16 data pairs of 2003 for model validation.

**Figure 5.**
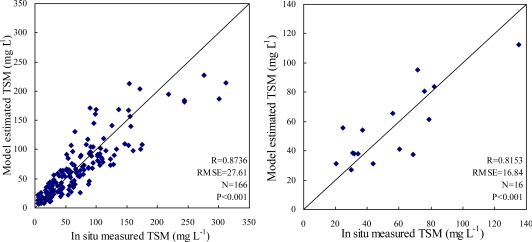
Comparison of *in situ* measured TSM and estimated TSM from the band normalized difference Cubic model listed in Table 2 using (**a**) 166 data pairs of 2005 for model development; (**b**) 16 data pairs of 2003 for model validation.

Table 2 lists the best fitting regression models between TSM concentration (mg L^−1^) and MODIS single band, band difference, band ratio and band normalized difference, showing that the last two models performed better than the first two models in terms of R and standard error. Comparison between *in situ* measured TSM and model-estimated TSM from the last two models using both the 166 data pairs for model development and the 16 data pairs for model validation is shown in Figures 4–5. This reveals that the Cubic model of band normalized difference between band 3 and band 1 performed a little better and so was selected as the best fitting retrieval model.

**Figure 6.**
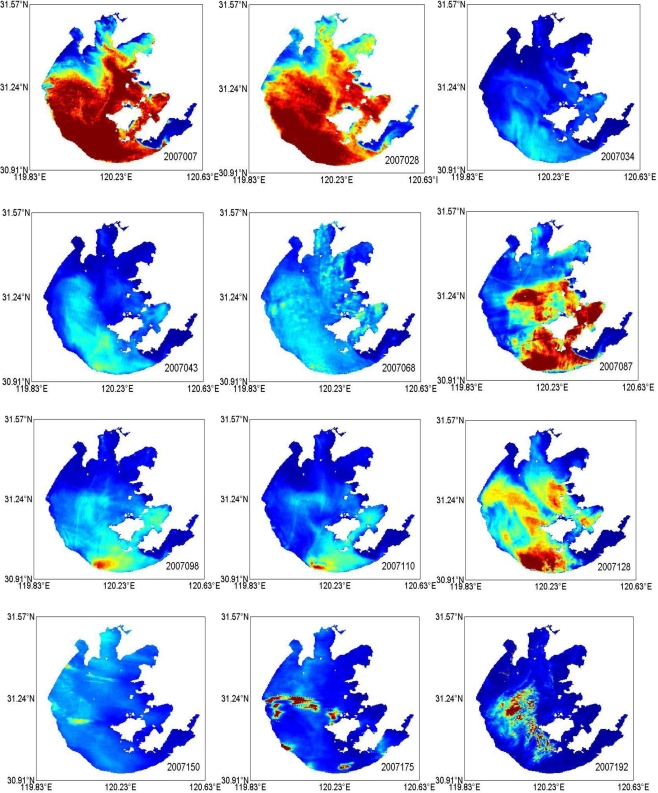

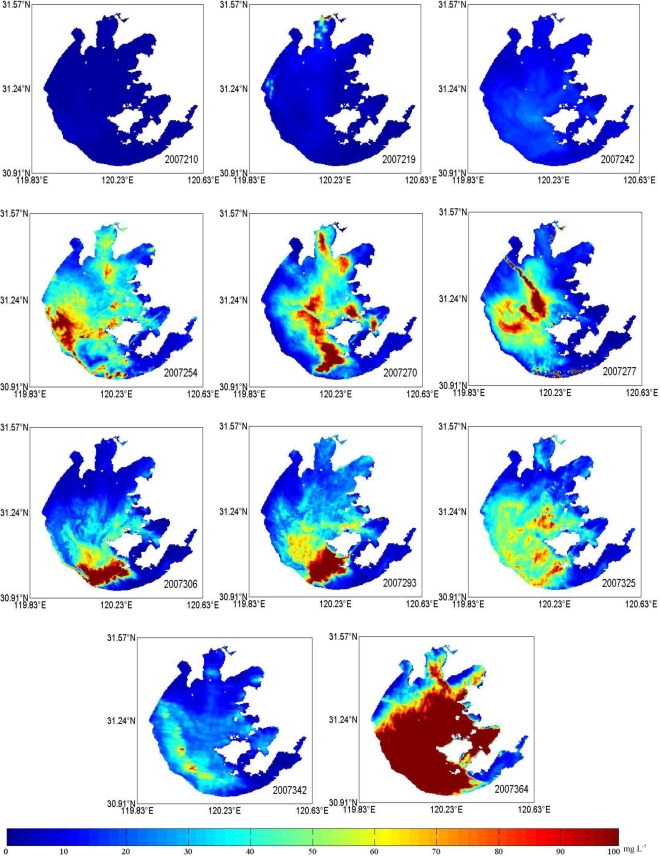
Distribution of TSM concentration (mg L^−1^) from January to December, 2007 in Lake Taihu from MODIS images.

Finally, the synoptic maps of TSM concentrations in 2007 were obtained (see Figure 6), which represent the distribution and variation of TSM concentrations in both temporal and spatial scales.

**Figure 7.**
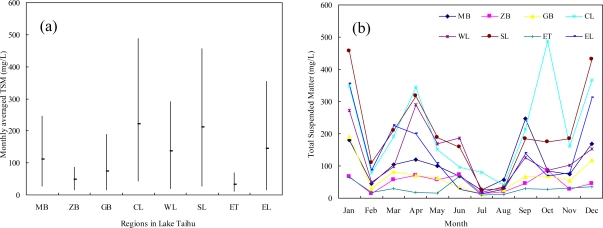
(**a**) Spatial variations in TSM in each region of Lake Taihu, 2007; (**b**) Temporal variations in TSM in each region of Lake Taihu, 2007.

The water body of Lake Taihu is divided into eight water regions. These include Meiliang Bay (MB), Zhushan Bay (ZB), Gonghu Bay (GB), Central Lake (CL), Western Lakeshore (WL), Southern Lakeshore (SL), East Taihu (ET) and Eastern Lakeshore (EL) (Figure 2). Figure 7 shows the distribution of TSM derived from MODIS images. A clear geographical and seasonal variation is visible. Figure 7(a) displays monthly averaged TSM concentrations in eight regions of Lake Taihu. Central Lake and Southern Lakeshore are characterized by extremely high TSM concentrations with annual averaged TSM concentrations being >200 mg L^−1^; Meiliang Bay, Western and Eastern Lakeshore also show relatively high TSM concentrations; while there is good water quality in East Taihu and Zhushan Bay, with the maximum TSM concentrations being <100 mg L^−1^. Moreover, the variation ranges of TSM concentration in the open areas are much larger than in the bays, and the minimum variation range is located in East Taihu due to the sheltered environment and dense hydrophytes. Furthermore, obvious seasonal variations in TSM are also characterized in the lake. Figure 7(b) shows the temporal variation in TSM in eight regions of Lake Taihu. It can be seen that on the whole, maximum TSM concentrations appear in winter, minimum in summer, with lower concentration peaks arising in spring (April represented) and autumn (September represented).

## 5. Discussion

### 5.1. MODIS Bands Retrieving TSM

A significant (R = 0.8712, n = 166) relationship was observed between TSM concentration (mg L^−1^) and relatively corrected MODIS Terra band 1 data used in this study (Table 2). The relationship is consistent with the conclusions of other studies [25–27]. A common method is to relate remotely sensed data measured in the red portion of the visible spectrum (ca. 600–700 nm) to parameters of water column sediment or particulate matter concentration. MODIS band 1 provides coverage in the red spectral region (620–670 nm) at sensitivity sufficient for case II water studies. Therefore, the characteristics of MODIS band 1 data, such as its medium spatial resolution (250 m), red band reflectance, high sensitivity, and near daily coverage, suggest that these images may be well suited for examining suspended particulates in inland waters [25]. However, the results also indicate that the compositions of band 3 and band 1 show better performance with the TSM concentration than just band 1. The optical properties of turbid waters, such as Lake Taihu, are jointly affected by the absorption and backscattering of pure water, chlorophyll *a*, chromophoric dissolved organic matter (CDOM) and TSM [9]. When chlorophyll *a* concentration is >10 μg L^−1^, there are an absorption peak and a reflectance peak in 672 nm and 695 nm respectively [28]. And both of high-concentration TSM and CDOM could affect the visible region of spectrum. But CDOM and chlorophyll *a* strongly absorb spectrum in the blue region (440 nm). Hence MODIS band 3 data (blue band reflectance) could assist band 1 data (red band reflectance) to weaken the influence from chlorophyll *a* and CDOM.

### 5.2. Model Performance Analysis

As shown in Figure 5, the model-estimated TSM was significantly related to the *in situ* measured TSM both in the model calibration and model validation phases, indicating that the empirical retrieval model developed in this study could map TSM concentrations relatively successfully. However, an underestimation also occurred, particularly when measured TSM concentrations were higher than 200 mg L^−1^ because of spectrum saturation effect (see Figure 4). Higher root mean square error (RMSE) of the estimated TSM may be caused by the following reasons: (1) water samples were collected from a fixed point while MODIS images give average radiance value over grid of 250 × 250 m; (2) time lag between field sampling and satellite overflight; (3) radiance value is influenced by adjacent pixels; (4) uncertainty of atmospheric correction; (5) the inherent error term in the statistical regression model.

### 5.3. Distribution of TSM in Lake Taihu in 2007

Previous studies both using remote sensing techniques [29] and *in situ* TSM measurements [30] also have demonstrated that the highest values of TSM concentrations in Lake Taihu appear in the Central Lake in winter and the lowest values appear in the East Taihu in summer. Average concentrations of TSM are 34.32 mg L^−1^ in the whole lake and 15.01–65.56 mg L^−1^ in different regions [30]. For Lake Taihu, TSM is composed of inorganic particles (e.g., suspended sands) and organic particles from plankton. Zhang *et al*. [30] found that only 30% of TSM in Lake Taihu came from organic particles. Hence inorganic particles are the key matter deciding TSM concentration [31]. As a large shallow lake, wind-induced sediment re-suspension has a significant contribution to TSM concentrations in Lake Taihu [32]. High wind speed causes high TSM concentrations in winter and in the Central Lake. A range of 5–6 m s^−1^ is always considered as the critical wind speed for sediment re-suspension [29,33], which means that little influence on sediment re-suspension could be found when wind speed is lower than 5 m s^−1^. Wind speed higher than 6 m s^−1^ could cause an obvious increase of TSM concentration. Inflowing rivers are another source of TSM in Lake Taihu [34]. However, the spatial resolution is only 250 m for MODIS images, so there is no obvious high concentration of TSM near the mouth of inflowing rivers.

Seasonal variations in TSM are notable in the Central Lake, but very weak in East Taihu. Because of the closed location and abundance of water plants, the TSM concentration in East Taihu is always lower than 50 mg L^−1^ and is steady during the whole year.

It should be noted that remote sensing images can only retrieve the TSM concentration at the water surface, and cannot show the vertical profiles of TSM. However, Lake Taihu is very shallow, and it has been found that TSM concentration of the lake changes only slightly with depth, except in areas near the inflowing rivers [35].

## 6. Conclusions

Monitoring water quality parameters is critical to many scientific and environmental studies. Satellites provide the only platform for acquiring repeated and frequent synoptic-scale observations over continental and global scales [4]. In this study, a common TSM retrieval model of Lake Taihu was tentatively developed, based on a relative atmospheric correction-PIFs method, which integrates the procedures of Du [23] and Chen [24]. The distribution characteristics of TSM concentrations in 2007 were analyzed using this model and MODIS images.

The common model retrieving TSM concentration showed that a significant (R = 0.8736, n = 166) relationship was observed between TSM concentrations (mg L^−1^) measured *in situ* and relatively corrected MODIS Terra band 1 data, which is consistent with the conclusions of other studies. The model performance analysis indicated that this empirical model could map TSM concentrations successfully except when TSM concentration is higher than 200 mg L^−1^. The temporal and spatial distribution of TSM concentrations in Lake Taihu from January to December 2007 revealed that Central Lake and Southern Lakeshore are characterized by extremely high TSM concentrations, and there is good water quality in East Taihu. The variation range of TSM concentration in the open area is much larger than that in the bays, affected by higher wind velocities in the open area. The minimum variation range is located in East Taihu due to the sheltered environment and dense hydrophytes. On the whole maximum TSM concentrations appear in winter and the minimum appear in summer. This pattern is notable in the Central Lake, but very weak in East Taihu.

It should be noted that the approach developed in this study could be applied to other inland regions, but the specific relationship between TSM concentration and MODIS data (*i.e.*, the slope of the regression line, Table 2) may vary as a consequence of the content, type and particle size distribution of suspended matter.

## Figures and Tables

**Table 1.** Information of TSM field measurements and MODIS images in 2005.

**Date of observations**	**Number of samples**	**Concentration scope of TSM (mg L^−1^)**	**Date of MODIS image**
Jan 17	13	17.74–94.36	Jan 17
Feb 20 to 22	28	11.35–311.40	Feb 20
Apr 15	13	10.40–146.08	Apr 15
May 19 to 20	13	10.96–108.30	May 22
Jul 14	13	4.32–88.68	Jul 16
Aug 18	28	5.23–133.40	Aug 15
Sep 14 to 15	13	18.92–128.80	Sep 16
Oct 13 to 14	11	24.60–63.16	Oct 11
Nov 18	21	6.54–153.95	Nov 21
Dec 14 to 16	13	5.48–174.60	Dec 14

**Table 2.** Best fitting regression models between total suspended matter (TSM) concentrations and normalized radiance (base: January 2005, Taihu Lake area) of 250 m single band, band difference, band ratio and band normalized difference of moderate resolution imaging spectrometer (MODIS).

**Retrieval Models**	**R**	**s.e.**	**F**
*Ln(TSM)*= 0.015 × *b*1 + 0.003 ×*b*1^2^ −0.282	0.8712	0.439	256.310
$\text{Ln}(\text{TSM})=166.960\times \frac{1}{b3-b1}-2.192$	0.7906	0.546	272.919
$\text{Ln}(\text{TSM})=-16.997\times \frac{b3}{b1}+3.326\times {\left(\frac{b3}{b1}\right)}^{2}+23.681$	0.8725	0.417	292.994
$\text{Ln}(\text{TSM})=-29.707\times (\frac{b3-b1}{b3+b1})+41.886\times {\left(\frac{b3-b1}{b3+b1}\right)}^{3}+11.358$	0.8736	0.418	291.665

s.e. standard error of the estimate, N = 166, P < 0.001; bn is band n.
